# *catena*-Poly[[bis­(1-ethyl-1*H*-imidazole-κ*N* ^3^)cadmium]-di-μ-chlorido-[(1-ethyl-1*H*-imidazole-κ*N* ^3^)cadmium]-di-μ-chlorido-[(1-ethyl-1*H*-imidazole-κ*N* ^3^)cadmium]-di-μ-chlorido-[bis­(1-ethyl-1*H*-imidazole-κ*N* ^3^)cadmium]]

**DOI:** 10.1107/S160053681104894X

**Published:** 2011-11-30

**Authors:** Qian Xu, Run-Qiang Zhu

**Affiliations:** a Ordered Matter Science Research Center, College of Chemistry and Chemical Engineering, Southeast University, Nanjing 211189, People’s Republic of China

## Abstract

The asymmetric unit of the crystal structure of the title compound, [Cd_2_Cl_4_(C_5_H_8_N_2_)_3_]_*n*_, contains two Cd^II^ cations, three 1-ethyl-1*H*-imidazole ligands, and four Cl^−^ anions. The two Cd^II^ atoms have quite different coordination environments: one is octa­hedrally coordinated by four Cl atoms and two N atoms from two 1-ethyl-1*H*-imidazole ligands, and the second is in a severely distorted fivefold coordination by four Cl atoms and one N atom from a 1-ethyl-1*H*-imidazole ligand. Adjacent Cd^II^ cations are inter­connected alternately by pairs of chloride bridges, generating an infinite step-like chain along the *a* axis. One ethyl group of the 1-ethyl-1*H*-imidazole ligand is disordered over two sets of sites with a 0.668 (13):0.332 (13) site-occupancy ratio.

## Related literature

For general background to compounds with organic framework structures and with ferroelectric properties, see: Ye *et al.* (2009 ▶); Zhang *et al.* (2009 ▶).
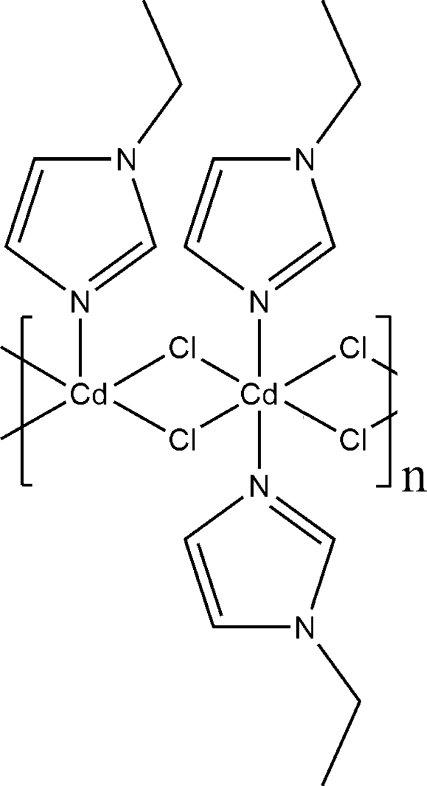


## Experimental

### 

#### Crystal data

[Cd_2_Cl_4_(C_5_H_8_N_2_)_3_]
*M* *_r_* = 655.02
Monoclinic, 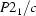

*a* = 15.227 (3) Å
*b* = 8.8651 (18) Å
*c* = 18.069 (4) Å
β = 110.34 (3)°
*V* = 2286.9 (8) Å^3^
*Z* = 4
Mo *K*α radiation
μ = 2.34 mm^−1^
*T* = 293 K
0.30 × 0.25 × 0.20 mm

#### Data collection

Rigaku SCXmini diffractometer
Absorption correction: multi-scan (*CrystalClear*; Rigaku, 2005 ▶) *T*_min_ = 0.501, *T*_max_ = 0.626
23098 measured reflections
5241 independent reflections
4674 reflections with *I* > 2σ(*I*)
*R*_int_ = 0.027

#### Refinement

*R*[*F* ^2^ > 2σ(*F* ^2^)] = 0.028
*wR*(*F* ^2^) = 0.059
*S* = 1.17
5241 reflections
251 parameters
33 restraints
H-atom parameters constrained
Δρ_max_ = 0.51 e Å^−3^
Δρ_min_ = −0.78 e Å^−3^

### 

Data collection: *CrystalClear* (Rigaku, 2005 ▶); cell refinement: *CrystalClear*; data reduction: *CrystalClear*; program(s) used to solve structure: *SHELXS97* (Sheldrick, 2008 ▶); program(s) used to refine structure: *SHELXL97* (Sheldrick, 2008 ▶); molecular graphics: *DIAMOND* (Brandenburg & Putz, 1999 ▶); software used to prepare material for publication: *SHELXL97*.

## Supplementary Material

Crystal structure: contains datablock(s) I, global. DOI: 10.1107/S160053681104894X/vn2017sup1.cif

Structure factors: contains datablock(s) I. DOI: 10.1107/S160053681104894X/vn2017Isup2.hkl

Additional supplementary materials:  crystallographic information; 3D view; checkCIF report

## Figures and Tables

**Table 1:** Selected bond lengths (Å)

Cd1—N1	2.250 (2)
Cd1—N3	2.267 (2)
Cd1—Cl1	2.6259 (11)
Cd1—Cl1^i^	2.6995 (8)
Cd1—Cl2	2.7203 (8)
Cd1—Cl3	2.8930 (12)
Cd2—N5	2.227 (2)
Cd2—Cl3	2.4713 (8)
Cd2—Cl4	2.5120 (8)
Cd2—Cl2	2.6340 (11)
Cd2—Cl4^ii^	2.7526 (11)

Symmetry codes: (i) 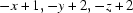; (ii) 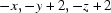.

**Fig. 1.**
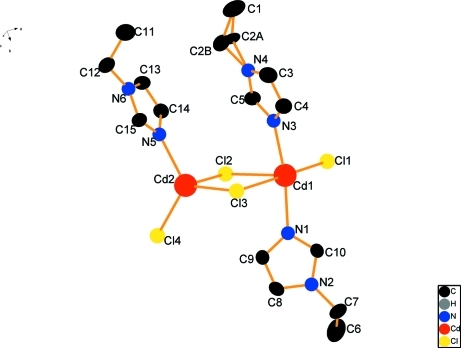
: Labeling scheme of (I). Thermal displacement ellipsoids depicted at 50% probability.

**Fig. 2.**
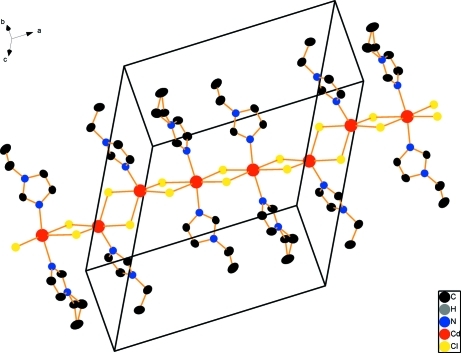
: Packing diagram of the title compound.

## supplementary crystallographic information

### Comment

The title compound, [C_15_H_24_Cd_2_Cl_4_N_6_], (I), was prepared from 1-ethyl-1*H*-imidazole and cadmium(II) chloride in *N*,*N*-dimethylformamide. The X-ray crystal structure of the title complex at 298 K (Fig. 1) shows a novel infinite one-dimensional coordination chain along the *a* axis (Fig. 2). There are two types of Cd atoms with different coordination environments: Cd(1) is coordinated by two 2-ethyl imidazole ligands and four bridging Cl atoms, and Cd(2) is in severely distorted pentahedral coordination by four bridging Cl atoms, and one 2-ethyl imidazole ligand.

In order to examine possible structure phase transitions of compound (I), we measured its temperature-dependent dielectric constant. Large dielectric anomalies usually indicate structural changes such as paraelectric-to-ferroelectric phase transitions. Unfortunately, the dielectric constant of compound (I) goes smoothly in the temperature range 93–273 K, suggesting the absence of distinct phase transitions (Ye *et al.*, 2009; Zhang *et al.*, 2009).

### Experimental

A mixture of CdCl2 (2.27 g, 10 mmol) and 2-ethyl imidazole (1.82 g, 20 mmol) in water was stirred for several minutes at room temperature, to which was then added 10 ml *N*,*N*-dimethylformamide. Colourless block-shaped crystals suitable for X-ray diffraction analysis were obtained by slow evaporation of the solution at room temperature over 2 weeks.

### Refinement

Positional parameters of all H atoms except for H1A, H1B and H1C were calculated geometrically and the H atoms were set to ride the C atoms to which they are bonded, with *U*_iso_(H) = 1.2 *U*_iso_(C) and 1.5*U*_iso_(C) for methyl H atoms. The positional parameters of the H atoms (C1) were refined freely. And in the last stage of the refinement, they were restrained with the C—H = 0.96 (2)Å with *U_iso_*(H)=1.5*U_iso_*(C).

### Crystal data

**Table:**

[Cd_2_Cl_4_(C_5_H_8_N_2_)_3_]	*F*(000) = 1280
*M**_r_* = 655.02	*D*_x_ = 1.893 Mg m^−^^3^
Monoclinic, *P*2_1_/*c*	Mo *K*α radiation, λ = 0.71073 Å
Hall symbol: -P 2ybc	Cell parameters from 5246 reflections
*a* = 15.227 (3) Å	θ = 2.8–27.5°
*b* = 8.8651 (18) Å	µ = 2.34 mm^−^^1^
*c* = 18.069 (4) Å	*T* = 293 K
β = 110.34 (3)°	Block, colourless
*V* = 2286.9 (8) Å^3^	0.30 × 0.25 × 0.20 mm
*Z* = 4	

### Data collection

**Table:**

Rigaku SCXmini diffractometer	4674 reflections with *I* > 2σ(*I*)
Radiation source: fine-focus sealed tube	*R*_int_ = 0.027
graphite	θ_max_ = 27.5°, θ_min_ = 3.0°
ω scans	*h* = −19→19
Absorption correction: multi-scan (*CrystalClear*; Rigaku, 2005)	*k* = −11→11
*T*_min_ = 0.501, *T*_max_ = 0.626	*l* = −23→23
23098 measured reflections	2 standard reflections every 150 reflections
5241 independent reflections	intensity decay: none

### Refinement

**Table:**

Refinement on *F*^2^	Primary atom site location: structure-invariant direct methods
Least-squares matrix: full	Secondary atom site location: difference Fourier map
*R*[*F*^2^ > 2σ(*F*^2^)] = 0.028	Hydrogen site location: inferred from neighbouring sites
*wR*(*F*^2^) = 0.059	H-atom parameters constrained
*S* = 1.17	*w* = 1/[σ^2^(*F*_o_^2^) + (0.0209*P*)^2^ + 1.5151*P*] where *P* = (*F*_o_^2^ + 2*F*_c_^2^)/3
5241 reflections	(Δ/σ)_max_ = 0.002
251 parameters	Δρ_max_ = 0.51 e Å^−^^3^
33 restraints	Δρ_min_ = −0.78 e Å^−^^3^

### Special details

**Table:**

Geometry. All e.s.d.'s (except the e.s.d. in the dihedral angle between two l.s. planes) are estimated using the full covariance matrix. The cell e.s.d.'s are taken into account individually in the estimation of e.s.d.'s in distances, angles and torsion angles; correlations between e.s.d.'s in cell parameters are only used when they are defined by crystal symmetry. An approximate (isotropic) treatment of cell e.s.d.'s is used for estimating e.s.d.'s involving l.s. planes.
Refinement. Refinement of *F*^2^ against ALL reflections. The weighted *R*-factor *wR* and goodness of fit *S* are based on *F*^2^, conventional *R*-factors *R* are based on *F*, with *F* set to zero for negative *F*^2^. The threshold expression of *F*^2^ > σ(*F*^2^) is used only for calculating *R*-factors(gt) *etc*. and is not relevant to the choice of reflections for refinement. *R*-factors based on *F*^2^ are statistically about twice as large as those based on *F*, and *R*- factors based on ALL data will be even larger.

### Fractional atomic coordinates and isotropic or equivalent isotropic displacement parameters (Å^2^)

**Table:**

	*x*	*y*	*z*	*U*_iso_*/*U*_eq_	Occ. (<1)
C1	0.2535 (3)	0.3210 (4)	0.7812 (2)	0.0622 (10)	
H1A	0.2185	0.2300	0.7790	0.093*	
H1C	0.3122	0.2969	0.7754	0.093*	
H1B	0.2185	0.3873	0.7394	0.093*	
C2A	0.2704 (3)	0.3936 (4)	0.8556 (2)	0.042 (3)	0.332 (13)
H2A	0.3278	0.3539	0.8935	0.050*	0.332 (13)
H2B	0.2197	0.3693	0.8744	0.050*	0.332 (13)
C2B	0.2259 (6)	0.4184 (8)	0.8346 (5)	0.066 (2)	0.668 (13)
H2C	0.2360	0.3650	0.8837	0.079*	0.668 (13)
H2D	0.1595	0.4400	0.8112	0.079*	0.668 (13)
C3	0.2872 (2)	0.6581 (4)	0.79499 (18)	0.0450 (7)	
H3A	0.2728	0.6360	0.7418	0.054*	
C4	0.3207 (2)	0.7902 (3)	0.83155 (17)	0.0398 (7)	
H4A	0.3333	0.8755	0.8070	0.048*	
C5	0.3075 (2)	0.6412 (3)	0.91979 (18)	0.0417 (7)	
H5A	0.3090	0.6020	0.9680	0.050*	
C6	0.5076 (3)	1.5520 (6)	1.2355 (2)	0.0877 (16)	
H6A	0.5458	1.6406	1.2514	0.132*	
H6B	0.5439	1.4645	1.2586	0.132*	
H6C	0.4550	1.5595	1.2530	0.132*	
C7	0.4739 (2)	1.5390 (4)	1.1487 (2)	0.0498 (8)	
H7A	0.4385	1.6288	1.1258	0.060*	
H7B	0.5273	1.5335	1.1313	0.060*	
C8	0.3308 (2)	1.3745 (3)	1.12864 (19)	0.0428 (7)	
H8A	0.2998	1.4343	1.1540	0.051*	
C9	0.3018 (2)	1.2413 (3)	1.09349 (19)	0.0421 (7)	
H9A	0.2465	1.1928	1.0906	0.050*	
C10	0.43245 (19)	1.2905 (3)	1.08022 (17)	0.0374 (7)	
H10A	0.4858	1.2833	1.0665	0.045*	
C11	−0.0447 (3)	0.3094 (4)	0.7616 (2)	0.0550 (9)	
H11A	−0.0935	0.2562	0.7217	0.083*	
H11B	0.0016	0.2388	0.7921	0.083*	
H11C	−0.0160	0.3805	0.7369	0.083*	
C12	−0.0856 (2)	0.3921 (4)	0.81485 (19)	0.0451 (7)	
H12A	−0.1335	0.4613	0.7836	0.054*	
H12B	−0.1152	0.3200	0.8391	0.054*	
C13	0.0617 (2)	0.4182 (3)	0.93522 (18)	0.0396 (7)	
H13A	0.0771	0.3166	0.9441	0.048*	
C14	0.10997 (19)	0.5361 (3)	0.97753 (17)	0.0365 (6)	
H14A	0.1649	0.5291	1.0211	0.044*	
C15	−0.00935 (19)	0.6269 (3)	0.88567 (16)	0.0336 (6)	
H15A	−0.0530	0.6937	0.8533	0.040*	
N1	0.36568 (15)	1.1879 (3)	1.06256 (14)	0.0337 (5)	
N2	0.41452 (16)	1.4056 (3)	1.12006 (14)	0.0339 (5)	
N3	0.33331 (15)	0.7790 (3)	0.91045 (13)	0.0334 (5)	
N4	0.2787 (2)	0.5636 (3)	0.85209 (16)	0.0463 (6)	
N5	0.06491 (15)	0.6681 (2)	0.94594 (13)	0.0321 (5)	
N6	−0.01384 (16)	0.4769 (3)	0.87701 (14)	0.0339 (5)	
Cd1	0.369012 (13)	0.97139 (2)	0.998629 (12)	0.03133 (6)	
Cd2	0.109824 (13)	0.90222 (2)	0.986382 (12)	0.02988 (6)	
Cl1	0.53919 (5)	0.88774 (8)	1.08530 (4)	0.03651 (15)	
Cl2	0.27753 (4)	0.82083 (8)	1.08130 (4)	0.03515 (15)	
Cl3	0.17898 (5)	1.05852 (8)	0.90744 (4)	0.03683 (15)	
Cl4	0.06876 (5)	1.00365 (9)	1.10005 (4)	0.03945 (16)	

### Atomic displacement parameters (Å^2^)

**Table:**

	*U*^11^	*U*^22^	*U*^33^	*U*^12^	*U*^13^	*U*^23^
C1	0.084 (3)	0.043 (2)	0.057 (2)	−0.0094 (19)	0.021 (2)	−0.0090 (17)
C2A	0.058 (7)	0.018 (4)	0.054 (6)	−0.007 (4)	0.024 (5)	−0.003 (4)
C2B	0.077 (5)	0.055 (4)	0.087 (5)	−0.031 (3)	0.053 (4)	−0.030 (3)
C3	0.0518 (18)	0.0498 (18)	0.0333 (15)	0.0009 (15)	0.0147 (14)	−0.0061 (14)
C4	0.0474 (17)	0.0365 (16)	0.0372 (16)	0.0016 (13)	0.0167 (14)	0.0028 (13)
C5	0.0579 (19)	0.0358 (16)	0.0404 (16)	−0.0077 (14)	0.0283 (15)	−0.0069 (13)
C6	0.082 (3)	0.118 (4)	0.072 (3)	−0.053 (3)	0.037 (2)	−0.048 (3)
C7	0.0517 (19)	0.0323 (16)	0.064 (2)	−0.0108 (14)	0.0185 (17)	−0.0093 (15)
C8	0.0355 (15)	0.0426 (18)	0.0537 (19)	0.0034 (13)	0.0200 (14)	−0.0109 (14)
C9	0.0312 (15)	0.0424 (17)	0.0555 (19)	−0.0057 (13)	0.0188 (14)	−0.0137 (15)
C10	0.0304 (14)	0.0373 (16)	0.0479 (17)	−0.0011 (12)	0.0178 (13)	−0.0074 (13)
C11	0.062 (2)	0.058 (2)	0.0451 (19)	−0.0062 (17)	0.0177 (17)	−0.0156 (16)
C12	0.0444 (17)	0.0395 (17)	0.0491 (18)	−0.0096 (14)	0.0131 (15)	−0.0090 (14)
C13	0.0464 (17)	0.0247 (14)	0.0499 (18)	0.0041 (12)	0.0195 (15)	0.0022 (13)
C14	0.0339 (14)	0.0331 (15)	0.0413 (15)	0.0063 (12)	0.0116 (13)	0.0026 (13)
C15	0.0373 (15)	0.0268 (14)	0.0361 (15)	0.0006 (11)	0.0119 (12)	0.0023 (11)
N1	0.0282 (11)	0.0316 (13)	0.0418 (13)	−0.0014 (9)	0.0129 (10)	−0.0061 (10)
N2	0.0337 (12)	0.0286 (12)	0.0377 (13)	−0.0014 (10)	0.0102 (10)	−0.0036 (10)
N3	0.0328 (12)	0.0329 (12)	0.0357 (13)	−0.0032 (10)	0.0135 (10)	−0.0038 (10)
N4	0.0614 (17)	0.0392 (14)	0.0496 (15)	−0.0139 (12)	0.0336 (14)	−0.0150 (12)
N5	0.0347 (12)	0.0251 (11)	0.0375 (12)	0.0001 (9)	0.0137 (10)	−0.0004 (10)
N6	0.0382 (13)	0.0267 (12)	0.0389 (13)	−0.0028 (10)	0.0161 (11)	−0.0040 (10)
Cd1	0.02946 (11)	0.02810 (11)	0.03840 (12)	−0.00269 (8)	0.01428 (9)	−0.00548 (8)
Cd2	0.02865 (10)	0.02620 (10)	0.03691 (11)	−0.00126 (8)	0.01409 (8)	−0.00228 (8)
Cl1	0.0308 (3)	0.0377 (4)	0.0418 (4)	−0.0001 (3)	0.0136 (3)	0.0090 (3)
Cl2	0.0298 (3)	0.0415 (4)	0.0343 (3)	0.0030 (3)	0.0114 (3)	0.0043 (3)
Cl3	0.0335 (3)	0.0341 (3)	0.0436 (4)	−0.0006 (3)	0.0143 (3)	0.0083 (3)
Cl4	0.0342 (3)	0.0471 (4)	0.0354 (4)	0.0105 (3)	0.0099 (3)	−0.0062 (3)

### Geometric parameters (Å, °)

**Table:**

C1—C2A	1.4306	C10—N1	1.318 (3)
C1—C2B	1.460 (7)	C10—N2	1.331 (4)
C1—H1A	0.9600	C10—H10A	0.9300
C1—H1C	0.9600	C11—C12	1.506 (4)
C1—H1B	0.9600	C11—H11A	0.9600
C2A—N4	1.515 (4)	C11—H11B	0.9600
C2A—H2A	0.9700	C11—H11C	0.9600
C2A—H2B	0.9700	C12—N6	1.472 (4)
C2B—N4	1.492 (6)	C12—H12A	0.9700
C2B—H2C	0.9700	C12—H12B	0.9700
C2B—H2D	0.9700	C13—C14	1.351 (4)
C3—C4	1.354 (4)	C13—N6	1.364 (4)
C3—N4	1.370 (4)	C13—H13A	0.9300
C3—H3A	0.9300	C14—N5	1.377 (4)
C4—N3	1.374 (4)	C14—H14A	0.9300
C4—H4A	0.9300	C15—N5	1.320 (3)
C5—N3	1.313 (4)	C15—N6	1.338 (3)
C5—N4	1.338 (4)	C15—H15A	0.9300
C5—H5A	0.9300	Cd1—N1	2.250 (2)
C6—C7	1.475 (5)	Cd1—N3	2.267 (2)
C6—H6A	0.9600	Cd2—N5	2.227 (2)
C6—H6B	0.9600	Cd1—Cl1	2.6259 (11)
C6—H6C	0.9600	Cd1—Cl1^i^	2.6995 (8)
C7—N2	1.469 (4)	Cd1—Cl2	2.7203 (8)
C7—H7A	0.9700	Cd1—Cl3	2.8930 (12)
C7—H7B	0.9700	Cd2—Cl3	2.4713 (8)
C8—C9	1.341 (4)	Cd2—Cl4	2.5120 (8)
C8—N2	1.365 (4)	Cd2—Cl2	2.6340 (11)
C8—H8A	0.9300	Cd2—Cl4^ii^	2.7526 (11)
C9—N1	1.364 (3)	Cd1—Cl1^i^	2.6995 (8)
C9—H9A	0.9300	Cd2—Cl4^ii^	2.7526 (11)
			
C2A—C1—C2B	27.5 (4)	C11—C12—H12B	109.2
C2A—C1—H1A	109.5	H12A—C12—H12B	107.9
C2B—C1—H1A	104.0	C14—C13—N6	106.7 (2)
C2A—C1—H1C	109.5	C14—C13—H13A	126.6
C2B—C1—H1C	134.2	N6—C13—H13A	126.6
H1A—C1—H1C	109.5	C13—C14—N5	109.2 (3)
C2A—C1—H1B	109.5	C13—C14—H14A	125.4
C2B—C1—H1B	87.1	N5—C14—H14A	125.4
H1A—C1—H1B	109.5	N5—C15—N6	111.5 (2)
H1C—C1—H1B	109.5	N5—C15—H15A	124.3
C1—C2A—N4	113.50 (16)	N6—C15—H15A	124.3
C1—C2A—H2A	108.9	C10—N1—C9	105.1 (2)
N4—C2A—H2A	108.9	C10—N1—Cd1	124.04 (18)
C1—C2A—H2B	108.9	C9—N1—Cd1	130.79 (19)
N4—C2A—H2B	108.9	C10—N2—C8	106.4 (2)
H2A—C2A—H2B	107.7	C10—N2—C7	126.3 (3)
C1—C2B—N4	113.2 (5)	C8—N2—C7	127.3 (3)
C1—C2B—H2C	108.9	C5—N3—C4	105.1 (2)
N4—C2B—H2C	108.9	C5—N3—Cd1	128.25 (19)
C1—C2B—H2D	108.9	C4—N3—Cd1	126.01 (19)
N4—C2B—H2D	108.9	C5—N4—C3	106.7 (3)
H2C—C2B—H2D	107.8	C5—N4—C2B	128.2 (4)
C4—C3—N4	106.2 (3)	C3—N4—C2B	123.6 (4)
C4—C3—H3A	126.9	C5—N4—C2A	118.6 (3)
N4—C3—H3A	126.9	C3—N4—C2A	132.1 (3)
C3—C4—N3	109.7 (3)	C2B—N4—C2A	26.4 (4)
C3—C4—H4A	125.2	C15—N5—C14	105.5 (2)
N3—C4—H4A	125.2	C15—N5—Cd2	127.30 (18)
N3—C5—N4	112.3 (3)	C14—N5—Cd2	127.19 (19)
N3—C5—H5A	123.9	C15—N6—C13	107.1 (2)
N4—C5—H5A	123.9	C15—N6—C12	126.2 (2)
C7—C6—H6A	109.5	C13—N6—C12	126.6 (2)
C7—C6—H6B	109.5	N1—Cd1—N3	163.71 (8)
H6A—C6—H6B	109.5	N1—Cd1—Cl1	97.38 (6)
C7—C6—H6C	109.5	N3—Cd1—Cl1	98.78 (6)
H6A—C6—H6C	109.5	N1—Cd1—Cl1^i^	90.14 (6)
H6B—C6—H6C	109.5	N3—Cd1—Cl1^i^	89.86 (6)
N2—C7—C6	112.5 (3)	Cl1—Cd1—Cl1^i^	82.49 (3)
N2—C7—H7A	109.1	N1—Cd1—Cl2	91.68 (6)
C6—C7—H7A	109.1	N3—Cd1—Cl2	88.48 (6)
N2—C7—H7B	109.1	Cl1—Cd1—Cl2	96.98 (3)
C6—C7—H7B	109.1	Cl1^i^—Cd1—Cl2	178.16 (2)
H7A—C7—H7B	107.8	N1—Cd1—Cl3	82.42 (6)
C9—C8—N2	106.9 (3)	N3—Cd1—Cl3	81.52 (6)
C9—C8—H8A	126.6	Cl1—Cd1—Cl3	177.86 (2)
N2—C8—H8A	126.6	Cl1^i^—Cd1—Cl3	99.63 (3)
C8—C9—N1	109.6 (3)	Cl2—Cd1—Cl3	80.91 (3)
C8—C9—H9A	125.2	N5—Cd2—Cl3	118.48 (6)
N1—C9—H9A	125.2	N5—Cd2—Cl4	117.80 (6)
N1—C10—N2	112.0 (2)	Cl3—Cd2—Cl4	123.25 (3)
N1—C10—H10A	124.0	N5—Cd2—Cl2	94.36 (6)
N2—C10—H10A	124.0	Cl3—Cd2—Cl2	91.04 (3)
C12—C11—H11A	109.5	Cl4—Cd2—Cl2	91.50 (3)
C12—C11—H11B	109.5	N5—Cd2—Cl4^ii^	88.06 (6)
H11A—C11—H11B	109.5	Cl3—Cd2—Cl4^ii^	92.16 (3)
C12—C11—H11C	109.5	Cl4—Cd2—Cl4^ii^	82.98 (3)
H11A—C11—H11C	109.5	Cl2—Cd2—Cl4^ii^	174.48 (2)
H11B—C11—H11C	109.5	Cd1—Cl1—Cd1^i^	97.51 (3)
N6—C12—C11	111.9 (3)	Cd2—Cl2—Cd1	94.22 (3)
N6—C12—H12A	109.2	Cd2—Cl3—Cd1	93.68 (3)
C11—C12—H12A	109.2	Cd2—Cl4—Cd2^ii^	97.02 (3)
N6—C12—H12B	109.2		

Symmetry codes: (i) −*x*+1, −*y*+2, −*z*+2; (ii) −*x*, −*y*+2, −*z*+2.
